# Understanding the role of P2X7 in affective disorders—are glial cells the major players?

**DOI:** 10.3389/fncel.2015.00258

**Published:** 2015-07-08

**Authors:** Leanne Stokes, Sarah J. Spencer, Trisha A. Jenkins

**Affiliations:** ^1^School of Medical Sciences, Health Innovations Research Institute, RMIT UniversityMelbourne, VIC, Australia; ^2^School of Pharmacy, University of East AngliaNorwich, UK; ^3^School of Health Sciences, Health Innovations Research Institute, RMIT UniversityMelbourne, VIC, Australia

**Keywords:** P2X7, depression, microglia, inflammation, mouse models, SNP

## Abstract

Pathophysiology associated with several psychiatric disorders has been linked to inflammatory biomarkers. This has generated a theory of major depressive disorders as an inflammatory disease. The idea of pro-inflammatory cytokines altering behavior is now well accepted however many questions remain. Microglia can produce a plethora of inflammatory cytokines and these cells appear to be critical in the link between inflammatory changes and depressive disorders. Microglia play a known role in sickness behavior which has many components of depressive-like behavior such as social withdrawal, sleep alterations, and anorexia. Numerous candidate genes have been identified for psychiatric disorders in the last decade. Single nucleotide polymorphisms (SNPs) in the human P2X7 gene have been linked to bipolar disorder, depression, and to the severity of depressive symptoms. P2X7 is a ligand-gated cation channel expressed on microglia with lower levels found on astrocytes and on some neuronal populations. In microglia P2X7 is a major regulator of pro-inflammatory cytokines of the interleukin-1 family. Genetic deletion of P2X7 in mice is protective for depressive behavior in addition to inflammatory responses. P2X7^−/−^ mice have been shown to demonstrate anti-depressive-like behavior in forced swim and tail suspension behavioral tests and stressor-induced behavioral responses were blunted. Both neurochemical (norepinephrine, serotonin, and dopamine) and inflammatory changes have been observed in the brains of P2X7^−/−^ mice. This review will discuss the recent evidence for involvement of P2X7 in the pathophysiology of depressive disorders and propose mechanisms by which altered signaling through this ion channel may affect the inflammatory state of the brain.

## Inflammation as a Theory for Pathogenesis of Mood-Related Behavioral Changes

Recent evidence suggests that activation of inflammatory responses may contribute to the pathogenesis of affective disorders such as depression, bipolar disorders and schizophrenia. Results from studies of clinical psychosis indicate high levels of pro-inflammatory macrophage-derived cytokines in both serum and plasma in psychotic relative to normal patients. Much of the available research to date has focused on circulating levels of pro-inflammatory agents, including the key interleukins (particularly IL-6, IL-2, IL-1β), tumor necrosis factor (TNF-α) and its receptors, and interferon gamma (IFN-γ) in schizophrenia (Gaughran, 2002; Drzyzga et al., 2006), bipolar disorder (O’Brien et al., 2006; Hope et al., 2009) and depression (Hiles et al., 2012; Liu et al., 2012; Dahl et al., 2014). However there is also increasing evidence of inflammatory cytokines and activation of microglia in the brains of affective disorder subjects. In schizophrenic patients elevated levels of IL-6 (Garver et al., 2003; Sasayama et al., 2013) and IL-1β cytokines (Miller et al., 2011) have been observed in cerebrospinal fluid (CSF); while in bipolar disorder an increase in IL-1β in the CSF of patients was recently found (Söderlund et al., 2011). Cytokines, in turn, promote detrimental neuronal consequences through activation of neuro-inflammatory cascades involving microglial release of oxidative species. Several post-mortem studies have reported an increased density of microglia in the hippocampus (Bayer et al., 1999) and regions of the prefrontal, cingulate and temporal cortices (Bayer et al., 1999; Radewicz et al., 2000; Steiner et al., 2008). Indeed, imaging and histological studies also report increased levels astrocyte and microglial markers (e.g., glial fibrillary acidic protein, GFAP, CD11b) in the prefrontal cortex (Rao et al., 2010) and hippocampus (Haarman et al., 2014) in bipolar patients and increased microglial priming and macrophage recruitment in the cingulate cortex of depressed brains (Torres-Platas et al., 2014). Moreover, in CSF collected from depressive patients, increases in inflammatory mediators, such as kynurenine and quinolinic acid released from microglia, are correlated with increased IFN-α, soluble TNF-α receptor 2 and the chemokine monocyte chemo-attractant protein (MCP-1) in CSF (Raison et al., 2010). While there should also be consideration of the external influence of medications (lithium, antidepressants antipsychotics), age, and patient health status (such as metabolic syndrome or chronic pain) which can all have their own inflammatory influences, it is still clear that elevated immune-inflammatory signaling activity is important in the etiology of mood disorders.

## Sickness Behavior; Inflammation-Related Behavioral Changes

The origin of pro-inflammatory cytokines found in the brain is yet to be clearly elucidated. While cytokines can be produced centrally, it is also of consideration that they are released by circulating immune cells and move to the brain through specific transport mechanisms such as via the circumventricular organs or across a dysfunctioning blood brain barrier. Evidence from animal studies indeed show that activation of the peripheral immune system influences brain pathology, cytokine levels, and behavior. Exposure to the bacterial endotoxin lipopolysaccharide (LPS) or the viral immunostimulant polyinosinic: polycytidylic acid (poly I:C) can induce sickness behavior, a depressive-like state where reductions in food consumption, body weight, social interaction and activity are observed (Yirmiya, 1996; Cunningham et al., 2007). This so-called sickness behavior is both a physical and psychological illness and is observed in humans, encompassing such complications as fatigue, lethargy, anhedonia, anorexia, and muscle and joint pain (Dantzer, 2009). Triggered by the production of pro-inflammatory cytokines by the immune system, it is thought to be part of a highly organized strategy to fight infections.

There are several glial (non-neuronal) cell populations in the brain, notably astrocytes, microglia and oligodendrocytes. The activation of brain microglia has been associated with depressive-like behavior in a number of recent studies (Tynan et al., 2010; Hinwood et al., 2012; Kreisel et al., 2014). In many cases psychological stress induced through prolonged restraint or unpredictable events, is demonstrated to alter microglial responses upregulating various cellular activation markers such as Iba-1, CD11b, and MHC Class II. The number and density of microglia in particular brain regions appears to be altered on the same time-scale as the behavioral changes. Dynamic changes in both number and activation status of microglia have been recently documented with acute increases in microglial number followed by a decline in number with more sustained (chronic) stress (Kreisel et al., 2014). Potentially, this early increased density of activated microglia could amplify pro-inflammatory signaling through the release of inflammatory and neurotoxic mediators such as cytokines. Blocking the initial activation with minocycline rescued the decline in microglial numbers and the behavioral change (Kreisel et al., 2014).

## P2X7 is an Ion Channel Regulating Inflammatory Signaling

P2X7 is a purinergic ion channel activated by the known danger signal molecule and neurotransmitter, ATP (Bartlett et al., 2014). Many immune cells express this purinergic ion channel including myeloid lineage cells such as macrophages and their brain-resident counterparts, microglia. P2X7 expression is not restricted to microglia in the brain, with astrocytes, oligodendrocytes and certain populations of neurons exhibiting low levels of expression (reviewed in Bartlett et al., 2014). Over the last decade research into understanding the role of this ion channel in immune cell responses has revealed a critical function in the regulation of cytokine secretion from macrophages and microglia. It is clear that activation of P2X7 is a major physiological stimulus for rapid secretion of IL-1 family cytokines from macrophages and microglia (Ferrari et al., 1997, 2006). Evidence for involvement of P2X7 activation in regulating other pro-inflammatory cytokine secretion is less robust although there is evidence for a role in IL-6 secretion from macrophages (Solle et al., 2001) and both TNF-α and IL-6 secretion from microglia (Shieh et al., 2014). In addition to cytokines P2X7 is involved in inflammatory prostaglandin secretion (Barberà-Cremades et al., 2012) and release of lysosomal proteases such as cathepsins (Lopez-Castejon et al., 2010).

Transgenic mice deficient in P2X7 (P2X7^−/−^) were generated over a decade ago and have aided our understanding of the physiological and pathophysiological roles of this ion channel. P2X7^−/−^ mice were originally demonstrated to display reduced cytokine responses (Solle et al., 2001) and subsequent studies have documented reduced inflammatory-related disorders such as anti-collagen induced arthritis (Labasi et al., 2002). *Ex vivo* macrophages isolated from P2X7^−/−^ mice display no secretion of IL-1β, IL-18 and IL-1α cytokines in response to *in* *vitro* priming and challenge with ATP (Pelegrin et al., 2008). Furthermore, intraperitoneal injection of LPS into P2X7^−/−^ mice caused a reduced febrile response compared to wild-type mice, which was restored upon injection of recombinant IL-1β (Barberà-Cremades et al., 2012). This suggests that P2X7−induced IL-1β secretion plays a key pyrogenic role *in vivo*. The behavioral effects of injected LPS were not investigated by Barberà-Cremades et al. (2012), however a separate study by Csölle et al. (2013b) has demonstrated that LPS-induced anhedonia measured using a sucrose preference test is reduced in P2X7^−/−^ mice. These studies suggest that inflammatory responses to LPS are reduced in P2X7 deficient mice.

## P2X7 and Microglial Responses

Early studies demonstrated that activation of P2X7 stimulated IL-1β cytokine release from mouse microglial cells (Ferrari et al., 1997). Experiments utilizing P2X7^−/−^ mice demonstrated that P2X7 was crucial for ATP-mediated IL-1β release from microglia and that P2X7 deficiency attenuated LPS-induced expression of both IL-1β and TNFα (Mingam et al., 2008). In addition to control of cytokines, P2X7 on microglial cells has been linked to glial cytotoxicity, with the induction of apoptotic cell death following prolonged stimulation (Bartlett et al., 2013). P2X7 on microglia can also induce cortical neuron cell injury in a co-culture model system, using a process not requiring direct cellular contact (Skaper et al., 2006). Microglia deficient in P2X7 or treated with a P2X7 antagonist did not induce neuronal toxicity in this study highlighting a key role for this ion channel in secreting mediators capable of reducing neuronal viability (Skaper et al., 2006).

**Figure 1:**
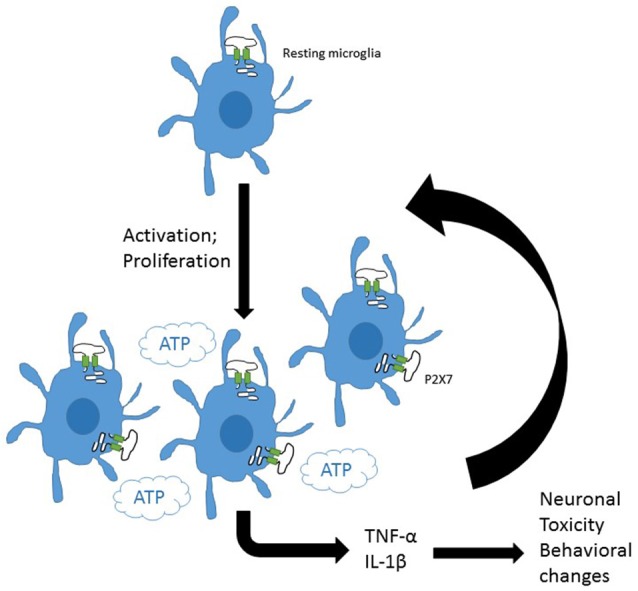
**P2X7 on central glial cells may drive inflammation.** Resting microglia express purinergic receptors including P2X7. Induction of microglial activation and proliferation increases microglial density in the brain. P2X7 is involved in both activation and proliferation of microglia. The presence of ATP, the major agonist for P2X7 channels would drive release of pro-inflammatory mediators from central microlia. These could amplify activation and proliferation of glia and induce toxic damage to neurons and neuronal signaling pathways potentially driving behavioral changes.

More recently P2X7 has been implicated in cellular proliferative responses in microglia amongst other cell types, with a study reporting that overexpression of P2X7 was sufficient to drive proliferation and activation of microglia (Monif et al., 2009). These observations are complemented by a study investigating basal and TNFα cytokine-induced proliferation in hippocampal-entorhinal slices where P2X7 was demonstrated to play a key role in proliferation (Zou et al., 2012). The involvement of P2X7 in microglial responses is summarized in Figure 1.

## P2X7 Deficient Mice are Resilient to Depressive-like Behavior

Four studies by different groups have so far investigated the effect of genetic deletion of P2X7 on behavior using standard depression tests in mice, namely the forced swim test and tail suspension test. All four studies demonstrated a significant reduction in immobility time in P2X7^−/−^ mice, a feature associated with anti-depressive behavior (Basso et al., 2009; Boucher et al., 2011; Csölle et al., 2013a, b). Differences between genotypes were more obvious after repeated challenge or stress suggesting that the demonstrated resilience is more pronounced to stressor-induced behavioral changes. Pharmacological blockade of P2X7 was also effective in altering depressive-like behavior and LPS-induced sickness behavior in mice (Csölle et al., 2013a, b). Selective, centrally penetrating P2X7 modulators may have potential for further development as therapies for CNS disorders including affective disorders. Three P2X7 antagonists A-438079, JNJ-47965567, and JNJ-42253432, have recently been demonstrated to attenuate amphetamine-induced behavioral sensitization in rodents (Bhattacharya et al., 2013; Gubert et al., 2014; Lord et al., 2014), a key process underlying motivational behavior. Moreover antidepressant and anxiolytic like behavior is observed after P2X7 antagonism in animal models exhibiting depressive-like symptomatology in forced swim and social interaction paradigms (Pereira et al., 2013; Lord et al., 2014; Wilkinson et al., 2014).

A major finding from the work by Csölle et al. (2013a) was that peripheral immune cells such as infiltrating monocytes or macrophages do not appear to be involved in protection from depressive behavior associated with P2X7 deficiency. To demonstrate this they used bone marrow chimera experiments. Here mice were irradiated to destroy circulating immune cells and underwent a bone marrow transplant from donor mice which were either wild-type (P2X7+) or knockout (P2X7−). Reconstituted mice with both P2X7 expressing and P2X7 deficient bone marrow demonstrated no difference in immobility time in the tail suspension test. The study concluded that deficiency of P2X7 only in peripheral immune cells gave no protection from depressive-like behavior (Csölle et al., 2013a). Whilst this study also suggested central microglia were not involved through the same bone marrow chimera approach, more data would be helpful to strengthen this particular conclusion. For example, does central injection of IL-1β restore depressive-like behavior in the P2X7^−/−^ mouse? This would indicate whether cytokines were driving depressive behavior. In addition, these behavioral studies use C57BL/6 mice which are known to carry a single nucleotide polymorphism (SNP) in the C-terminus of P2X7 affecting downstream signaling pathways including cytokine secretion (Adriouch et al., 2002). It would be interesting and relevant to determine if this effect is maintained in mouse strains carrying a high functioning P2X7.

Significant differences in expression (both up- and down-regulation) of genes involved in synaptic signaling were found by whole genome microarrays in the amygdala of P2X7^−/−^ mice (Csölle et al., 2013a). Moreover, exposure to restraint stress caused an acute elevation in adrenocorticotropic hormone (ACTH) and corticosterone levels in wild-type C57BL/6 mice but this was reduced in P2X7^−/−^ mice (Csölle et al., 2013a). This suggests that P2X7 deficiency may blunt the stress hormone response. The anterior pituitary, which is responsible for the production of stress hormones amongst others, expresses P2X channels (Koshimizu et al., 2000; Zemkova et al., 2006) and ATP is known to modulate ACTH secretion. Therefore it is possible P2X7 may play a role in the amplification of secretagogue signals from the hypothalamus.

## A Mutant P2X7 Allele Linked to Depression and Bipolar Disorder in Human Studies

There have been a number of genetic studies over the last decade linking a SNP in the human *P2RX7* gene to depression, anxiety, and bipolar disorder (Barden et al., 2006; Lucae et al., 2006; McQuillin et al., 2009; Soronen et al., 2011). Inheritance of the minor allele of this SNP (rs2230912-G) has also been correlated to severity of depression in diabetic and psychiatric patients (Hejjas et al., 2009; Nagy et al., 2008; Halmai et al., 2013). In contrast there are several genetic studies that do not find an association with *P2RX7* genotype at this polymorphic site and bipolar disorder, major depression or schizophrenia (Green et al., 2009; Viikki et al., 2011). A recent meta-analysis also suggested no association (Feng et al., 2014). Addressing the functional relevance of this SNP *in vitro* studies on circulating immune cells isolated from humans carrying the rs2230912-G allele have demonstrated that this genetic variant of P2X7 is inherited on a gain-of-function allele (Stokes et al., 2010). Importantly human monocytes expressing this rs2230912-G variant secreted more IL-1β in response to activation of P2X7 than monocytes expressing a wild-type variant (Stokes et al., 2010). Therefore one could speculate that microglia in the brain may also display enhanced cytokine secretion in response to P2X7 ion channel activation. Whilst this is difficult to demonstrate in the human system, a transgenic mouse approach knocking-in this human variant of P2X7 could begin to address this question.

In transgenic mouse studies genetic deletion of P2X7 eliminates a full-length P2X7 protein and protects against depressive learned helplessness behavior (Basso et al., 2009; Boucher et al., 2011; Csölle et al., 2013a). Could this also be true for humans? There are numerous loss-of-function SNPs in the human P2X7 gene which are demonstrated to have dramatic effects on receptor trafficking or receptor function (Sluyter and Stokes, 2011). Several studies have included some (but not all) loss-of-function P2X7 SNPs in their analysis but no difference in genotype frequency has so far been documented (Barden et al., 2006; Hansen et al., 2008).

## The Gliotransmitter ATP and Depressive Behavior

The purinergic signaling system is extensive in the body involving many receptors, ion channels and enzymes. ATP is a known co-transmitter released at synapses and extra-synaptic sites modulating the responses of glial and neurons (Burnstock, 2008). A recent study by Cao et al. presents a novel case for astrocyte-derived ATP as a rapid anti-depressant neurotransmitter (Cao et al., 2013). But how does this idea fit with a role for P2X7 (and potentially other purinergic receptors) in depressive disorders? Cao et al. (2013) suggest that astrocytes release ATP in the medial prefrontal cortex where the P2X2 receptor seems to be the downstream target for this anti-depressant action of ATP as evidenced by shRNA experiments. Astrocytes and microglia both express P2X7, which can be activated by relatively high concentrations of ATP (>100 μM); levels that may not be achieved in the brain where nucleotidases are widely expressed (Robson et al., 2006). Thus astrocyte-derived ATP may not reach concentrations high enough to stimulate pro-inflammatory signaling through P2X7. In P2X7^−/−^ mice, which demonstrate an antidepressant-like phenotype, Csölle et al. (2013a) demonstrated an up-regulation of a number of genes in the amygdala including *P2RX2* (11-fold change). Together with the evidence that immune cells (including microglia) do not affect the behavioral phenotype, perhaps this highlights a role for astrocytes, ATP, and P2X2, in the antidepressant effect of P2X7 deficiency? It is clear that more studies are required to understand the role of purinergic signaling in psychiatric disorders.

## Future Perspectives

In humans a correlation between high functioning P2X7 and affective disorders is often observed but this may be limited to patients with an inflammatory component, for example diabetic patients. Perhaps understanding more about the clinical features associated with affective disorders will lead to a useful stratification of these disorders and co-morbidity with other diseases. The evidence for involvement of P2X7 in depressive disorders from animal studies is promising but it is clear that more information about this ion channel and its role in inflammation and behavior is needed. Microglia are known to play a role in stress-induced depressive behavior but is P2X7 a major driver of inflammatory signaling in central microglia under conditions of chronic stress? P2X7 is not restricted to microglia and it is likely that astrocytes participate in regulation of microglial and neuronal responses. Future studies will hopefully address these issues and begin to determine whether this ion channel could be a novel drug target for affective disorders.

## Conflict of Interest Statement

The authors declare that the research was conducted in the absence of any commercial or financial relationships that could be construed as a potential conflict of interest.
